# Prevalence, incidence, and age at diagnosis in Marfan Syndrome

**DOI:** 10.1186/s13023-015-0369-8

**Published:** 2015-12-02

**Authors:** Kristian A. Groth, Hanne Hove, Kasper Kyhl, Lars Folkestad, Mette Gaustadnes, Niels Vejlstrup, Kirstine Stochholm, John R. Østergaard, Niels H. Andersen, Claus H. Gravholt

**Affiliations:** Department of Cardiology, Aarhus University Hospital, DK-8200 Aarhus N, Denmark; Department of Molecular Medicine, Aarhus University Hospital, Palle Juul-Jensens Boulevard 99, DK-8200 Aarhus N, Denmark; Department of Clinical Genetics, Copenhagen University Hospital, Rigshospitalet, DK-2100 Copenhagen, Denmark; The RAREDIS Database, Section of Rare Diseases, Department of Clinical Genetics, Copenhagen University Hospital, Rigshospitalet, DK-2100 Copenhagen, Denmark; Department of Cardiology, Rigshospitalet, DK-2100 Copenhagen, Denmark; Department of Endocrinology, Odense University Hospital, DK-5000 Odense C, Denmark; Institute of Clinical Reasearch, University of Southern Denmark, DK-5000 Odense C, Denmark; Centre for Rare Diseases, Department of Paediatrics, Aarhus University Hospital, DK-8200 Aarhus N, Denmark; Department of Endocrinology and Internal Medicine, Aarhus University Hospital, DK-8000 Aarhus C, Denmark

**Keywords:** Epidemiology, Rare diseases, Aortic aneurism, Lens subluxation, Aortic dissection

## Abstract

**Background:**

Marfan syndrome is a genetic disorder with considerable morbidity and mortality. Presently, clinicians use the 2010 revised Ghent nosology, which includes optional genetic sequencing of the *FBN1* gene, to diagnose patients. So far, only a few studies based on older diagnostic criteria have reported a wide range of prevalence and incidence. Our aim was to study prevalence, incidence, and age at diagnosis in patients with Marfan syndrome.

**Method:**

Using unique Danish patient-registries, we identified all possible Marfan syndrome patients recorded by the Danish healthcare system (1977–2014). Following, we confirmed or rejected the diagnosis according to the 2010 revised Ghent nosology.

**Results:**

We identified a total of 1628 persons with possible Marfan syndrome. We confirmed the diagnosis in 412, whereof 46 were deceased, yielding a maximum prevalence of 6.5/100,000 at the end of 2014. The annual median incidence was 0.19/100,000 (range: 0.0–0.7) which increased significantly with an incidence rate ratio of 1.03 (95 % CI: 1.02–1.04, *p* < 0.001). We found a median age at diagnose of 19.0 years (range: 0.0–74). The age at diagnosis increased during the study period, uninfluenced by the changes in diagnostic criteria. We found no gender differences.

**Conclusion:**

The increasing prevalence of Marfan syndrome during the study period is possibly due to build-up of a registry. Since early diagnosis is essential in preventing aortic events, diagnosing Marfan syndrome remains a task for both pediatricians and physicians caring for adults.

## Background

Since the first description of Marfan syndrome (MFS), decades of research in the syndrome [1] have contributed to the knowledge about the phenotypical presentation and the genetic background. In 1986, the definition of MFS described by the Berlin criteria [2], was purely based on the clinical phenotype. Later on, Dietz et al. found a connection between MFS and *FBN1*, the gene coding for the fibrillin protein [3]. The first Ghent criteria from 1996 (Ghent-I) [4], which were a revision of the Berlin criteria, used the newly discovered *FBN1* mutations as a component in the diagnostic criteria. In 2010, the revised Ghent criteria (Ghent-II) [5] highlighted *FBN1* mutation, aortic dilatation and ectopia lentis as cornerstones in the MFS diagnosis [5].

The most frequently quoted prevalence of MFS is 20/100,000 [6, 7]. The source is an early version of the textbook of Emery and Rimoins: Principles and practice of Medical Genetics [8], but the latest version only refers to a crude calculation of 4–6/100,000 based on MFS patients found in the catchment area of Johns Hopkins Hospital in Baltimore. During the last 70 years, only five studies report MFS prevalence, all but one based on the Berlin criteria. In 1958, Lynas et al. reported a prevalence of 1.5/100,000 in a population from Northern Ireland [9]. Sun et al. reported a prevalence of 17.2/100,000 in China in 1990 [10]. Gray et al. [11] reported a prevalence of 6.8/100,000 in the north-east Scottish population. A Danish study from 1997 by Fuchs et al. showed a prevalence of 4.6/100,000 [12]. Here the diagnosis was based on data from medical records and all cases were diagnosed before 1993. Chiu et al. in 2014 reported a much higher prevalence of 10.2/100,000, but the figures were solely based on data collected from 2000–2012 and without any regard to diagnostic criteria or clinical presentation [13]. Thus, there are no publications on the prevalence of clinically verified MFS based on the Ghent-I or the Ghent-II criteria and no prevalence studies report data including *FBN1* mutations. As the clinical manifestations of MFS may vary even within families with the same genetic background, it is not only difficult to diagnose MFS but also to assess the true prevalence of MFS based on clinical phenotyping of patients [14]. However, use of *FBN1* genotyping may represent a new dimension in diagnosing MFS and thereby provide a more accurate identification and classification of MFS [15].

Therefore, we set out to determine the prevalence and incidence of Marfan syndrome in Denmark using the current diagnostic approach as well as to describe the age diagnostic as a marker for the diagnostic delay in MFS defined as the time from birth until diagnosis.

## Methods

Since 1968, all Danish citizens have a unique personal identification number (CPR-number) in the Danish Central Person Register (www.cpr.dk) which is used in a number of Danish registers, thus providing a unique opportunity for record linkage, including The National Patient Register (NPR) [16] and The Danish Register of Cause of Death (DRCD) [17]. From 1977 and onwards, the NPR registered all in-patient contacts with the Danish healthcare system and from 1995, also registered all outpatient contacts. All contacts were given an International Classification of Diseases (ICD) code (ICD-8 until 1993 and ICD-10 from 1994 and onwards). DRCD record all death certificates since 1973 according to the ICD system, and used ICD-8 in 1973–1983, and ICD-10 from 1984 and onwards. DRCD was updated through 2013.

We retrieved CPR-numbers from all persons recorded in at least one of the two registries with the ICD-10 diagnosis Q87.4 “Marfan Syndrome” or ICD-8 759.80 “Arachnodactylia (syndroma Marfan)”.

As several persons were noted with an ICD-8 or ICD-10 diagnosis of MFS only based on the suspicion of suffering from MFS in the NPR register, all medical records were manually evaluated, to confirm or reject the diagnosis. As the MFS diagnosis has evolved significantly during the years with the changing criteria, Berlin [2], Ghent-I [4] and II [5], we decided to perform the medical record evaluation according to the Ghent-II criteria [5]. Medical records were accessed via a central electronic patient journal system (E-journal) provided by the Danish Healthcare System. If E-journal material was insufficient to determine whether the person had MFS or not, the original paper medical file was retrieved.

**Fig. 1:**
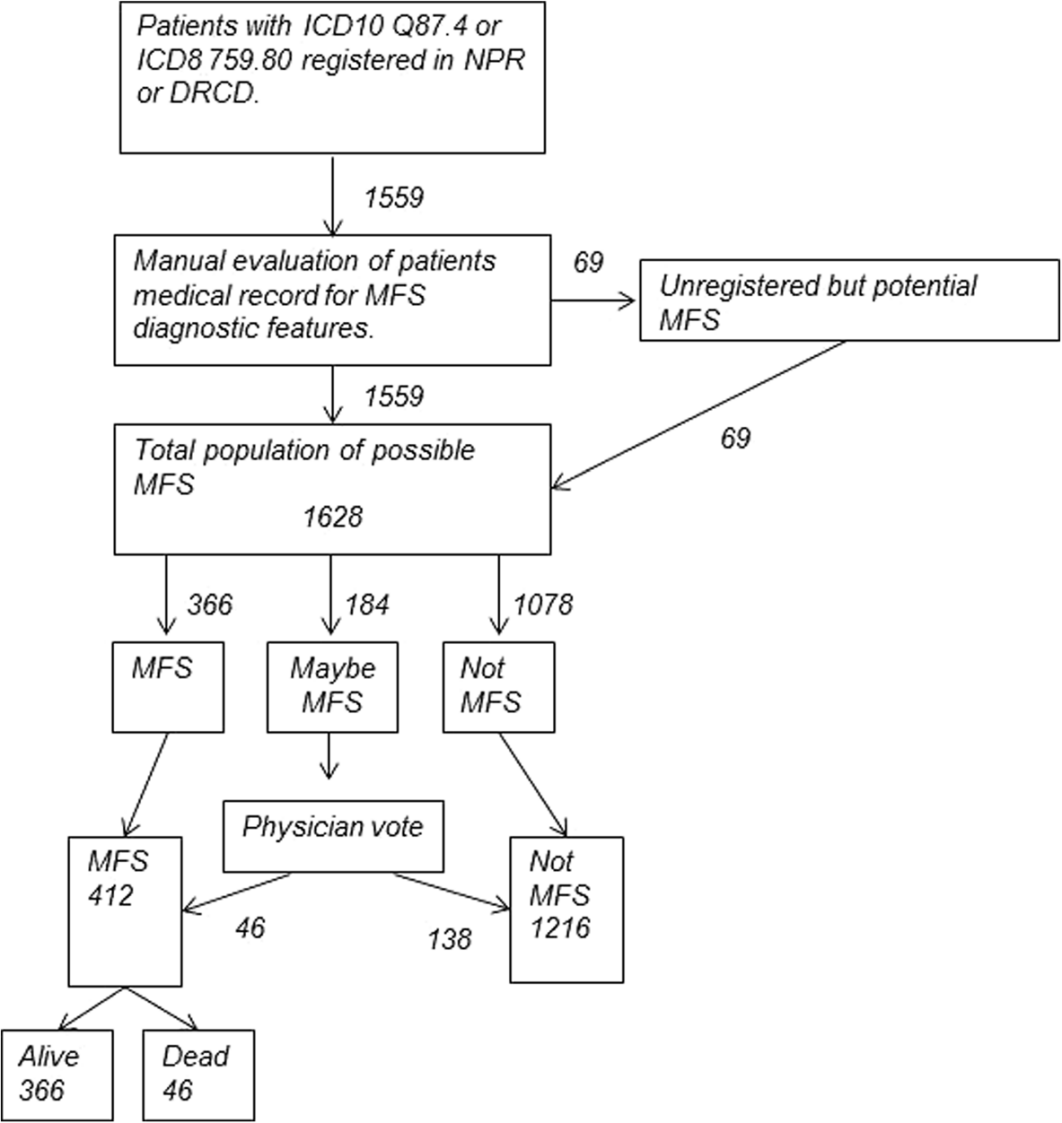
The total cohort and evaluation process defining patients with MFS

If we, during the evaluation, found other persons such as family members that could also have MFS, we evaluated their MFS status as well (Fig. 1).

There are seven ways a person can meet the Ghent-II criteria (Table 1). All who fulfilled at least one of the seven principal diagnostic features were classified as “MFS”, whereas all who did not fulfill any of the seven possible diagnostic criteria were classified as “not MFS”.

**Table 1:** The seven principal ways a person can meet the Ghent II criteria in the Marfan syndrome diagnosis

1) Ascending aorta dilatation^a^ & ectopia lentis	
2) Ascending aorta dilatation^a^ & a *FBN1* mutation	
3) Ascending aorta dilatation^a^ & minimum seven systematic points	
4) Ectopia lentis with a *FBN1* mutation known to cause ascending aorta dilatation^a^	
5) Family history of MFS & ectopia lentis	
6) Family history of MFS & minimum seven systematic points	
7) Family history of MFS & ascending aorta dilatation^a^	

^a^Or dissection of the ascending aorta

**Table 2:** Yearly incidence per 100,000 of Marfan syndrome in Denmark

Year of diagnosis	Male			Female			Total		
	Diagnosed	Population	Incidence	Diagnosed	Population	Incidence	Diagnosed	Population	Incidence
1977	6	2,513,000	0.24	1	2,567,000	0.04	7	5,079,879	0.12
1978	1	2,520,000	0.04	1	2,577,000	0.04	2	5,096,959	0.04
1979	1	2,526,000	0.04	4	2,586,000	0.15	5	5,111,537	0.10
1980	2	2,529,053	0.08	1	2,593,012	0.04	3	5,122,065	0.06
1981	0	2,528,225	0.00	2	2,595,764	0.08	2	5,123,989	0.04
1982	5	2,523,825	0.20	4	2,595,330	0.15	9	5,119,155	0.18
1983	3	2,521,220	0.12	1	2,595,244	0.04	4	5,116,464	0.08
1984	3	2,517,942	0.12	3	2,594,188	0.12	6	5,112,130	0.12
1985	3	2,517,072	0.12	4	2,594,036	0.15	7	5,111,108	0.14
1986	2	2,520,563	0.08	1	2,595,710	0.04	3	5,116,273	0.06
1987	1	2,526,020	0.04	1	2,598,774	0.04	2	5,124,794	0.04
1988	3	2,527,996	0.12	3	2,601,258	0.12	6	5,129,254	0.12
1989	5	2,528,165	0.20	1	2,601,613	0.04	6	5,129,778	0.12
1990	2	2,530,597	0.08	2	2,604,812	0.08	4	5,135,409	0.08
1991	3	2,536,391	0.12	7	2,610,078	0.27	10	5,146,469	0.19
1992	2	2,544,454	0.08	8	2,617,672	0.31	10	5,162,126	0.19
1993	8	2,554,594	0.31	5	2,626,020	0.19	13	5,180,614	0.25
1994	18	2,563,442	0.70	15	2,633,200	0.57	33	5,196,642	0.64
1995	9	2,573,324	0.35	9	2,642,394	0.34	18	5,215,718	0.35
1996	7	2,592,222	0.27	9	2,658,805	0.34	16	5,251,027	0.30
1997	5	2,604,937	0.19	3	2,670,184	0.11	8	5,275,121	0.15
1998	10	2,615,669	0.38	5	2,679,191	0.19	15	5,294,860	0.28
1999	7	2,625,421	0.27	6	2,688,156	0.22	13	5,313,577	0.24
2000	7	2,634,122	0.27	5	2,695,898	0.19	12	5,330,020	0.23
2001	7	2,644,319	0.26	3	2,704,893	0.11	10	5,349,212	0.19
2002	10	2,654,146	0.38	9	2,714,208	0.33	19	5,368,354	0.35
2003	8	2,662,423	0.30	15	2,721,084	0.55	23	5,383,507	0.43
2004	9	2,670,135	0.34	10	2,727,505	0.37	19	5,397,640	0.35
2005	5	2,677,292	0.19	6	2,734,113	0.22	11	5,411,405	0.20
2006	3	2,685,846	0.11	9	2,741,613	0.33	12	5,427,459	0.22
2007	4	2,696,662	0.15	5	2,750,422	0.18	9	5,447,084	0.17
2008	6	2,712,666	0.22	3	2,763,125	0.11	9	5,475,791	0.16
2009	7	2,732,020	0.26	4	2,779,431	0.14	11	5,511,451	0.20
2010	5	2,743,286	0.18	4	2,791,452	0.14	9	5,534,738	0.16
2011	10	2,756,582	0.36	3	2,804,046	0.11	13	5,560,628	0.23
2012	10	2,766,776	0.36	10	2,813,740	0.36	20	5,580,516	0.36
2013	9	2,778,852	0.32	9	2,823,776	0.32	18	5,602,628	0.32
2014	9	2,792,279	0.32	6	2,834,956	0.21	15	5,627,235	0.27
1977–2014	215	2,609,146	0.22	197	2,671,729	0.19	412	5,281,280	0.20

Data on gender only, with an accuracy of 1000 individuals for the years 1977–1979. For the line 1977–2014 the presented data are the summed number of patients diagnosed with Marfan syndrome, mean values for the Danish population and mean values for Marfan syndrome incidence

If medical records were insufficient (or non-existing) in both electronic and non-electronic versions, or if for some reason (ex. deceased or emigrated) it was not possible to fully determine the persons MFS status, a committee of three MFS specialist physicians evaluated the available persons data and determined the MFS status by consensus. All persons with no clinical data were classified as “not MFS”.

The study was approved by the Scientific Ethical Committee and the Danish Data Protection Agency.

### Statistical analysis

Age at diagnosis was studied by median age at diagnosis with range interval and time trends were studied with quantile regression including 95 % confidence intervals (CI). Time trends in incidence including 95 % confidence intervals (CI) were analyzed using Poisson regression. To graphically illustrate time trends in incidence we used linear regression lines. Gender difference and difference between the cohort with MFS and without MFS were studied using Mann-Whitney’s nonparametric test. *P <* 0.05 was considered significant. Stata 12.1 for Windows (StataCorp LP, College Station, TX, USA) was used for all calculations.

## Results

**Fig. 2:**
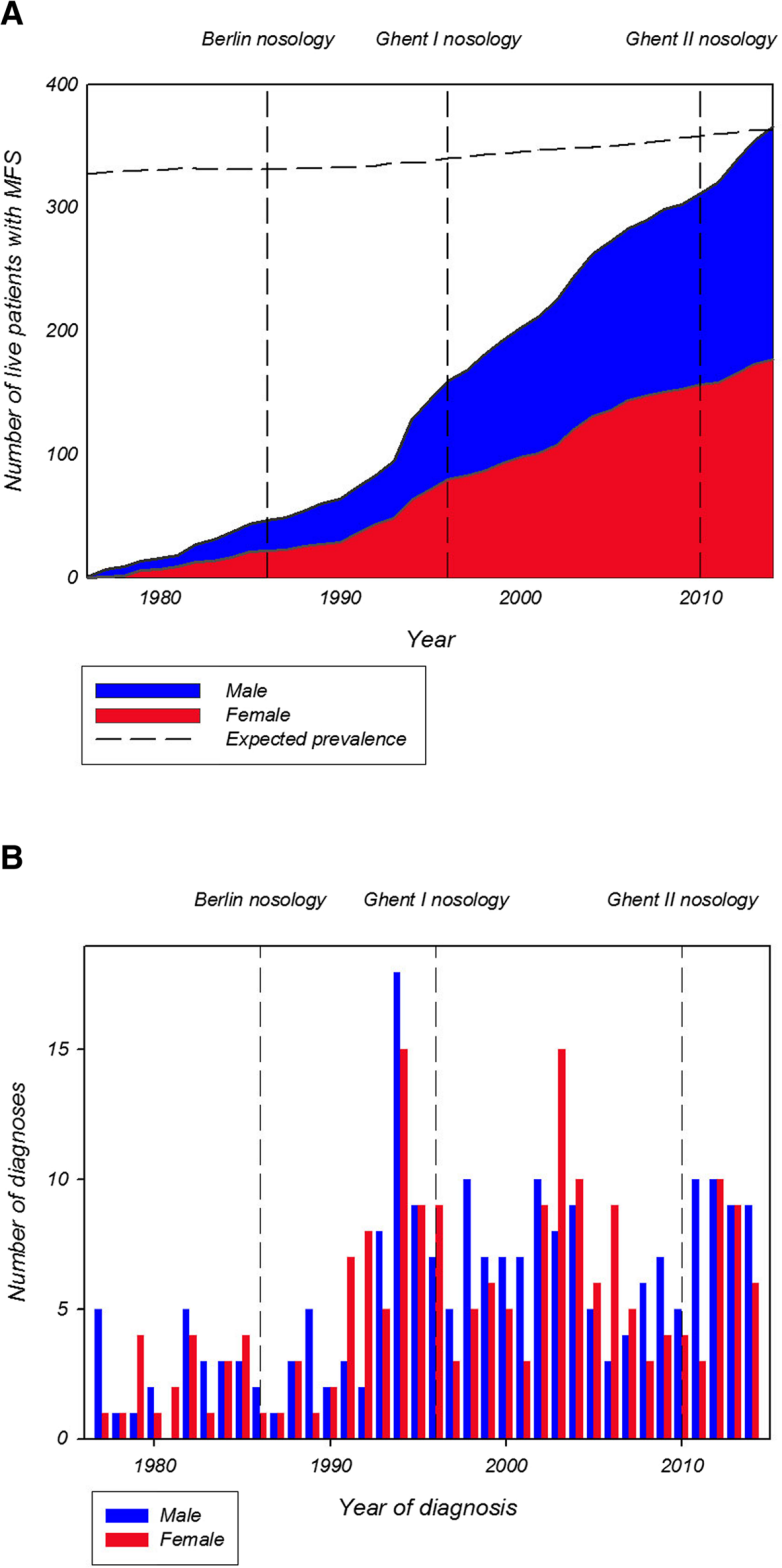
**a** Observed cumulated absolute number of Marfan syndrome patients alive per year during the study period from 1977 to 2014. The dashed line (expected prevalence) indicates the expected number of Marfan syndrome patients assuming a prevalence of 6.5 per 100,000 Danish inhabitants. The year of change of nosology is indicated by a horizontal line and marked with the nosology name. **b** Number of Marfan syndrome patients diagnosed per year during the study period from 1977 to 2014. Bars divided by sex. The year when the MFS nosology was changed, is indicated by a horizontal line and marked with the nosology name

From NPR and DRCD, we extracted all persons registered with a relevant ICD-8 or ICD-10 diagnosis, which resulted in 1559 unique CPR-numbers (Fig. 1). During the evaluation of their medical records, we found 69 additional potential MFS persons resulting in a total cohort of 1628. During the evaluation process, we found 22.5 % (*n* = 366) patients fulfilling one of the seven ways to obtain the MFS diagnosis and rejected 1078 cases (66.2 %). In 184 (11.3 %), it was not possible to accurately determine whether the persons fulfilled the diagnostic criteria. Thus, sufficient data was present in 1444 (88.7 %) of the total cohort. 73 (4.5 %) had no clinical data and were either deceased (*n* = 69) or emigrated (*n* = 4). They were classified as “not MFS”. A committee of three physicians specialized in MFS (KAG, NHA and CHG), evaluated every remaining case (*n* = 111) and reached consensus on their MFS status. Forty-six were determined to have MFS and the remaining 65 were registered as “not MFS”. Thus, 1216 (74.7 %) had “not MFS” and 412 (male *n* = 215) classified as “MFS”. Among the 412 classified as MFS, 366 (male *n* = 189) were still alive at the end of 2014 (Fig. 2a).

There was no difference in gender (*p* = 0.3) and birth year between persons classified with or without MFS (*p* = 0.2).

### Prevalence and incidence

As of January 1^st^ 2015, the population of Denmark was 5,659,715 inhabitants (www.dst.dk) yielding a point prevalence of MFS of 6.5/100,000. We also calculated an average prevalence increase of 0.17/100,000 per year during the study period. The average number of MFS diagnosed patients annually was 11.1 with a significantly increasing incidence during the study period (Fig. 2a, b).

**Fig. 3:**
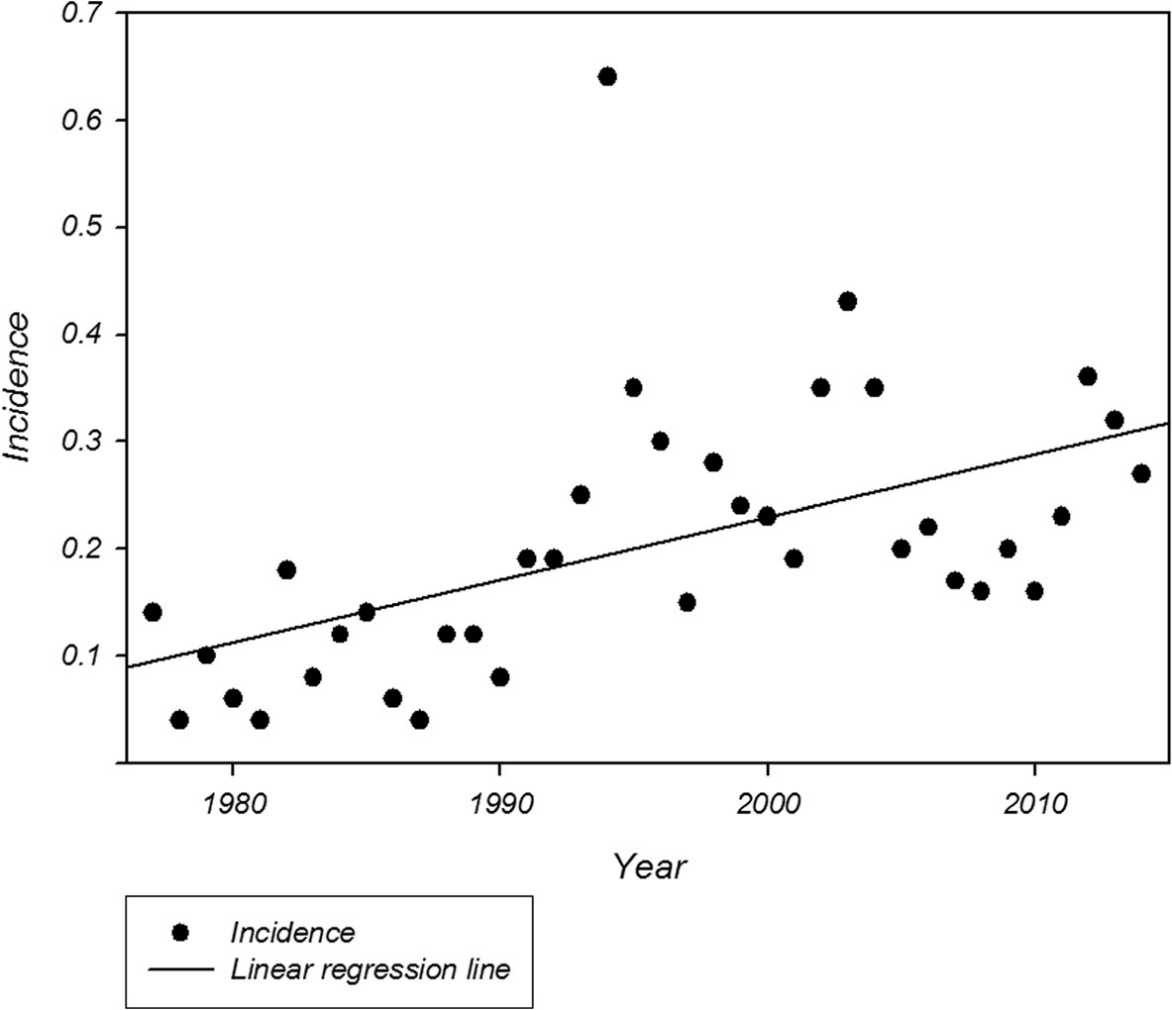
Yearly incidence of Marfan syndrome in Denmark during the study period 1977 to 2014. For clarity, the significant increase in incidence during the study period is visualized by linear regression

The median annual incidence was 0.19/100,000 (0.0–0.7) (Table 2). During the study period, the absolute number of patients diagnosed with MFS annually increased significantly with an incidence rate ratio (IRR) of 1.03 (95 % CI: 1.02–1.04, *p* < 0.001) (Fig. 3). Since this increase could be due to lack of access to patient records early in the study period, we calculated the IRR for the last 10 years of the study period (2004–2014) resulting in an increasing IRR of 1.11 (95 % CI 1.01–1.21 *p* = 0.018). We identified no difference in IRR between the two genders during the study period (*p* = 0.47).

**Fig. 4:**
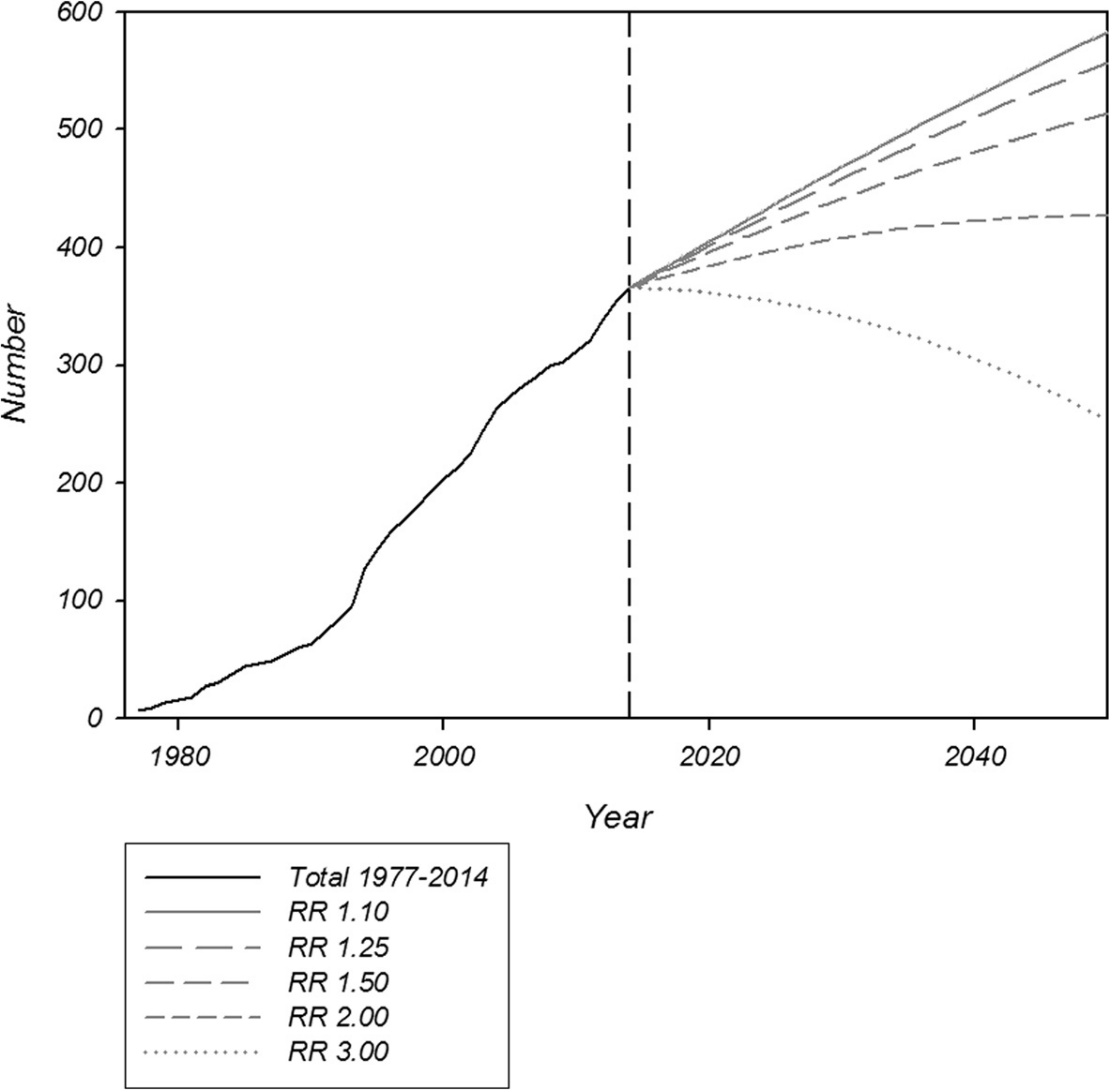
Absolute numbers of Marfan syndrome in Denmark during the study period 1977 to 2014 and the absolute theoretical numbers extrapolated onwards to 2050. Extrapolation is based on the expected Danish population according to Statistical Denmark (www.dst.dk). Incidence is set to 0.19 per 100,000 as found in this study. Since there has been no studies reporting mortality ratios in comparison with general population, we have for illustration plotted five different relative risks (RR) of mortality compared to the general Danish population

Based on the current prevalence of MFS in our data and exploring different scenarios with different relative risk of mortality of 1.1, 1.25, 1.5, 2.0 or 3.0 in comparison with the general population and using forecasts for the development of the Danish population, we generated future trajectories of the prevalence of MFS (Fig. 4).

### Age at diagnosis

**Fig. 5:**
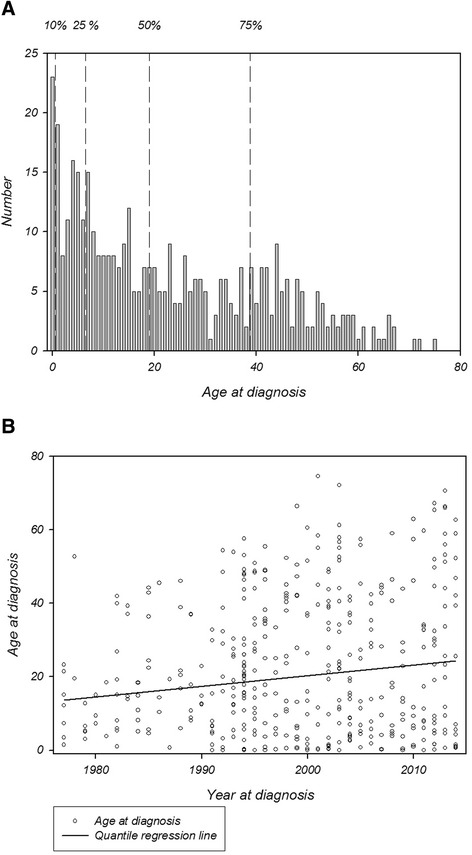
**a** Number of Marfan syndrome patients by age at diagnosis. Patients diagnosed during the study period 1977 to 2014. Dashed lines indicating the age when 10, 25, 50 and 75 % of MFS patients are diagnosed. **b** Age at diagnosis versus year of diagnosis during the study period 1977 to 2014. The non-significant increase in age at diagnosis is visualized by quantile regression

The median age at diagnosis for the entire MFS group was 19.0 (0.0–74.5) years. There was no difference in age at diagnosis between males and females (median age at diagnosis: males 18.3 years (0.0–74.5) and females 19.9 years (0.0–72.1) (*p* = 0.3)). By the age of 1.5 years 10 %, 6.5 years 25 % and 38.8 years 75 % of the entire cohort were diagnosed, respectively, but age at diagnosis extended into the seventies (Fig. 5a). There was a tendency towards an increasing age at diagnosis of 0.29 (95 % CI -0.03–0.60, *p* = 0.075) years per year of diagnosis during the study period (Fig. 5b).

### FBN1 evaluation

Of the total cohort of 412 MFS patient 196 had been tested for *FBN1* mutations, with 193 having a *FBN1* mutation known to cause MFS. In three cases no known mutation was found, however they fulfilled the Ghent-II nosology by other criteria (aorta ascendens dilatation and minimum seven systemic points (*n* = 2) or by a family history of MFS and aorta ascendens dilatation (*n* = 1)). One patient was only evaluated for FBN1 mutations and could have a MFS related disorder. One patient was evaluated with a wide genetic panel spanning all Marfan related disorders. One patient was evaluated for FBN1 mutations and collagen anomaly. However, since the three patients fulfilled the criteria for MFS, we included them in the study cohort.

### Preimplantation and prenatal diagnostics

Since 2000, only extremely few patients have chosen preimplantation diagnostics due to limited service and long waiting times. A total of 24 MFS patients chose prenatal diagnostics and of these, ten fetuses carried an FBN1 mutation. In only three cases did the parents choose an abortion before the 12^th^ gestation week, indicating that currently such low numbers of legal abortions are unlikely to affect the prevalence and incidence of MFS (unpublished data from the Danish Cytogenetic Central Register).

## Discussion

As the first study of MFS according to the Ghent-II nosology, this report shows a prevalence of MFS to be 6.5/100,000 in the uniform health care system in Denmark. We also find that the diagnosis of MFS is made throughout the entire lifetime, with only half of all diagnoses confirmed before the age of 19 years. Importantly, it seems as if the diagnostic vigilance is increasing during the study period, illustrated by the significant increase in incidence.

We identified the Danish MFS prevalence to be 41 % higher than the previously reported Danish prevalence of 4.6/100,000 published almost 20 years ago [12]. In the 1990’ies, patients were diagnosed according to the Berlin nosology and the study primarily focused on ectopia lentis [12], whereas the present study subjected every single patient file to close scrutiny including every aspect of MFS. Interestingly, the nosology of MFS changed three times (1986, 1996 or 2010) during the study period, but we did not see any changes in incidence or prevalence related to different diagnostic criteria (Fig. 2a, b).

It is difficult to estimate the true prevalence of MFS and we are well aware that some patients with MFS in Denmark still need to be diagnosed and identified. Based on the present data, we expect that the prevalence of MFS will increase by approximately 0.17 patients/100.000 the next many years. The reasons behind the imprecision are multifactorial – i.e. multiple factors exert an effect and some may tend to decrease and others may tend to increase the observed prevalence. Thus mortality, and to a lesser extent diagnostic practice, will influence the absolute number of MFS in the Danish population. Since the exact relative risk of death is not known in MFS, we have illustrated this with a set of different scenarios, where it can be appreciated that if the relative risk of death is below 2.0, we will continue to see an expanding population of MFS (Fig. 4). Newer literature seems to suggest that mortality is decreasing for contemporarily treated MFS [18, 19], which would obviously increase the prevalence, as illustrated in our future projections (Fig. 4). Another important component in the increasing prevalence is the build-up of a registry, where more patients are often diagnosed than censured (deceased or emigrated) in the beginning of the history of a registry. This phenomenon is seen in many other studies of rare syndromes [20, 21]. Moreover, our data also illustrate a significantly increasing incidence rate ratio, which was evident even during the last 10 years of the study period. This increase in incidence could be caused by an increased focus on the disease and better knowledge about the syndrome by healthcare professionals, resulting in more patients being diagnosed even at a high age. Better diagnostics and the increased use of genotyping could also explain the increasing incidence, as could more intense investigations of affected families, currently recommended in guidelines [5]. Factors expected to decrease prevalence, such as preimplantation diagnostics followed by induced abortions currently only seem to play a very minor role. On the other hand more surviving well-treated individuals with a disease causing MFS mutation could also lead to increased transmission of MFS mutations.

Since 1996 there have been two centers in Denmark handling rare diseases including MFS. We believe that the centralization of rare diseases has resulted in an increased focus on examining pedigrees of MFS families, and thereby diagnosing adult family members with MFS.

Given that MFS is a potentially life-threatening disorder due to aortic disease [22–24], an early diagnosis is important and will provide better overall health for the MFS patient [22, 25]. It is our impression that some physicians expect diagnosing MFS to be mainly a task for pediatricians. However, our data clearly indicate that many MFS patients are not diagnosed until late in life which means that all medical specialties should focus on even subtle clinical signs [26] and not hesitate to refer potential undiagnosed MFS patients, even from an elderly population. Early diagnosis should be the goal since this could reduce health expenditures and possibly avoid cases of dissection and sudden death [27]. The significant increase in age at diagnosis in the current cohort and especially the diagnosis of quite old individuals, may well illustrate diagnosis of less affected individuals, a factor that could also lead to an increased prevalence of the MFS.

Phenotyping patients can be difficult and time-consuming and clinical manifestations resulting in MFS will sometimes only be evident when the patient reaches adulthood and thereby “grows into the diagnosis”. Clinical manifestations may also vary considerably and some patients have a milder phenotype making it difficult to accurately assess the prevalence of MFS [28]. In theory, *FBN1* genotyping should help solve this problem, but discovery of the *FBN1*-gene did not seem to have any immediate effect on the age at diagnosis (Fig. 2a). However, of the 412 patients diagnosed with MFS in our study cohort approximately half of the population (*n* = 196) had been tested for *FBN1* mutations, even that it is a snapshot it may be the reason why genotyping did not have a major impact in this cohort. It is our impression that access to genetic sequencing is improving and we have not seen the full impact of *FBN1* screening on the prevalence of MFS. *FBN1* genotyping represents a new dimension in diagnosing MFS that could accelerate the process, but still some difficulties remain in the correct interpretation of *FBN1* gene test results [29].

### Strength and limitations

The present study is a nationwide register study, covering all subjects ever given a diagnosis of MFS. Furthermore, the study was performed in a uniform public healthcare system making it possible to report precise data on age at diagnosis. The rising incidence, prevalence and age at diagnosis during the study period could be due to information bias in the early time period of the study. Since Danish hospitals are only legally required to keep patient records 10 years after the latest entry, many hospitals have destroyed records. Nonetheless, most Danish hospital records are computerized and kept infinitely. Therefore, data collection from journals may not be as good in the beginning of the study period compared to the latest 10–15 years, resulting in some bias in interpretation of data over time. Many of the elderly persons registered in the first part of the study period are dead before computerization of records and for that reason their records were purely paper files and often not available for evaluation. Consequently, some persons had to be evaluated as “not MFS” due to lack of journal data, while they in reality might have suffered from MFS. This could obviously create a bias in the assessment of the median age at diagnosis and the prevalence early in the study period. However, this problem should not affect our data during the latter part of the study period.

## Conclusion

We found a MFS prevalence of 6.5/100,000 in the Danish population but expect a growing prevalence during the next years, since we saw an increasing prevalence and incidence during the study period. We also found a striking time span of patients age at diagnosis of zero to seventy-four years and a median age at diagnosis of 19.0 years emphasizing that diagnosing MFS is a task for both pediatricians as well as other clinicians.

### Ethics approval and consent to participate

The study was approved by the Scientific Ethical Committee of Region Midtjylland and the Danish Data Protection Agency.

## Abbreviations

CI: Confidence interval
CPR: Danish Central Personal Register
DRCD: The Danish Register of Causes of Death
FBN1: Fibrillin-1 gene
Ghent-I: First revised Ghent nosology [4]
Ghent-II: Second revised Ghent Nosology [5]
ICD: International classification of disease
IRR: Incidence rate ratio
MFS: Marfan Syndrome
NPR: The National Patient Register
RR: Relative risk
